# Editorial: Highlights in sport, leisure, tourism, and events: 2021/22

**DOI:** 10.3389/fspor.2023.1191051

**Published:** 2023-04-03

**Authors:** Vassilios Ziakas, Andrew Adams, Alana Thomson, Katie Schlenker

**Affiliations:** ^1^ Independent Researcher, Leeds, United Kingdom; ^2^ Bournemouth University, Poole, United Kingdom; ^3^ Federation University Australia, Ballarat, VIC, Australia; ^4^ University of Technology Sydney, Sydney, NSW, Australia

**Keywords:** physical activity, psycho-social impacts, social media, event portfolio, sport participation, public perceptions, sustainability, disruptive research

**Editorial on the Research Topic** Highlights in sport, leisure, tourism, and events: 2021/22

This special edition puts a spotlight on a diverse collection of papers that contribute important insights to our existing knowledge bases in the sport, leisure, tourism and events sectors. The range of insights highlight the value of, and need for, interdisciplinary research that crosses boundaries among these interconnected sectors (1, 2). The work presented also attests to the need for conceptual innovation in approaching problems if we are to disrupt and redefine new ways of thinking and doing to keep pace with the contemporary dynamics our applied fields face (3).

The topic of physical activity and its connection with school sport and sport events was evident in two articles. This is an important topic as we recalibrate post-COVID, and the contributions are timely in offering alternative approaches that attempt to disrupt the ways we approach physical activity in school sport and sport events. First, Teare and Taks focused on sport participation impacts from sport events. Through a review of literature, they identified that most studies tended to focus on mega sport events and adult populations, with the majority employing cross-sectional data and quantitative methods. They argued for: a need for more longitudinal studies, a stronger focus on youth populations, participant events, and smaller-sized events. The authors argue that these measures can (re)locate opportunities and means to derive further social value of sport events by creating an upswell in sport participation over the long term surrounding event hosting, and in doing so can advance the research agenda for sport participation outcomes from sport events.

Second, Carlton et al. examined participant physical activity levels during the most popular high school sports in the United States. Organized sports in school settings provide children and adolescents with opportunities to achieve recommended amounts of moderate to vigorous physical activity (MVPA). Carlton et al.'s empirical study identified specific sports and approaches to session facilitation which contributed most effectively to facilitating physical activity. Sports that emphasized game simulation, fitness, and skill development drills had higher levels of MVPA. The findings align with our theme of disruption by providing an important evidence base to inform policy and practice world-wide as it relates to school sport practice and increasing physical activity levels of young people.

The psycho-social impacts of sport and events was apparent in a further two articles which examined issues of soccer supporter violence, and the psychic income of youth residents concerning a football mega-event. While the psycho-social elements are much-cited in sport and event texts, and in the rhetoric promulgated by event proponents, these topics have been identified as under-researched and warranting further empirical studies (4). First, Lindstrom examined the psychological underpinnings of soccer supporter violence in a study of Swedish soccer supporters. The study found that honesty-humility negatively predicted violent intentions, and team identification predicted violent intentions. While collective narcissism was associated with violent intentions, when both honesty-humility and team identification were accounted for, collective narcissism did not predict violent intentions. Given the recent growth in attention we have seen on violence in leisure spaces (5, 6), and the increased expectation that leisure spaces will be safe and inclusive, Lindstrom's timely study provides important implications for the design of violence prevention interventions amongst soccer supporters and serves to disrupt the culture of violence that prevails in some aspects of sport.

The second article contributing to our understanding of psycho-social impacts is that by Ishac and Swart who studied youth residents' perceptions of hosting major international sport events. Using the 2019 IAAF Athletics World Championships in Doha, Qatar, they examine psychic income as the perceived emotional and psychological benefits residents receive from hosting an international sport event. They argue that for some nationals, perceived impact was higher, and the perceived impact was higher for females than males. Importantly, the authors identify that these events—whether attended in person or not—can still generate a positive psychic income. By evaluating the psychic income received by youth from different nationalities residing in Qatar, this study contributes to our theme of disruption by providing decision-makers and organizers with a better understanding of differential outcomes generated from hosting major international sport events, and how these can be leveraged going forward. The geographic and cultural make up of this study also presents a useful and timely challenge to dominant Western and Eurocentric research narratives concerning the social impacts and legacies of events (4).

The next two papers are about changing perceptions. The first paper by Radmann et al. focuses on how social media contributes to the development of people's perceptions, perceived knowledge and concept of authenticity around their sport (equestrian sport). They present the results of an empirical study exploring how social media influencers within equestrian sport communicate with their followers. The research examined the social media accounts of the six biggest equestrian influencers in Sweden and Norway in order to discover how ideas and perceived knowledge are formed and transferred in the sport. Findings indicate that the equestrian influencers build trust and intimacy and construct authenticity in relation to their sport. The relationship between influencers and followers centers on a shared love of horses which creates the intimacy needed as a base for other messages (perceived knowledge and advertisement). Perceived as experts, influencers are likely to impact followers' perception of the human-animal relationship as well as their consuming behavior, and together with their followers, they shape and determine what is considered as an authentic stable culture. Little research has been done on social media and equestrianism, and thus this research contributes to the larger body of work on social media and sport. It contributes to new ways of thinking, interpreting, and understanding influencer-follower dynamics, perceived authenticity and the impact on consumer behavior in the sport landscape.

The second looks at how participating in outdoor sports increases awareness of sustainability and helps develop a caring culture for the environment Trendafilova and Ziakas provide a conceptual analysis of the sport-nature-culture nexus and focus on the social aspects and interactions central in shaping attitudes, behaviors and cultures about outdoor sports and the environment. They offer a framework of a social sport ecology that synthesizes management, nature sports, neo-tribalism, and non-representation theoretical perspectives. This framework is predicated on the thesis that outdoor sports allow participants to feel more connected to nature, which may foster a desire to care for the natural world and contribute to environmentally sustainable practices. Design of the sports ecosphere therefore requires aligning the affective and cognitive dimensions of experience in ways that build caring cultures for the environment. This study offers important insights for future practice, with respect to the design of interventions for the environmental management of outdoor recreational spaces.

A final two articles are themed around the idea of maximizing the social value of events; one in relation to advancing the portfolio approach of events, and the other in relation to measuring and maximizing residents' social value gained from publicly-funded sport events. First, Ziakas provides a review paper focused on the emergent phenomenon of event portfolios. He proposes a new conceptual lens named “morphosynthesis” to explain the multilevel integration processes that shape event portfolios and enable the interlinking of social networks and the community through an array of events. Morphosynthesis offers a transdisciplinary perspective on the study of event portfolios that focuses on community engagement with event portfolios and their capacity to contribute to the strengthening of a host community's social fabric. This research contributes to our theme of disruption by highlighting the need to rethink traditional one-off event hosting approaches, and instead through the lens of morphosynthesis, consider the contributions an event portfolio can make to the social fabric of a host community.

Bakhsh et al. offer a further contribution to the theme of maximizing the social value of events. They investigated monetary valuation methods used to analyze residents' social value from hosting a publicly-funded major sport event, and tested the selected methods to determine residents' social value from hosting the 2010 Olympic Winter Games in Vancouver, Canada. Findings highlighted the importance of using both the reverse contingent valuation method and opportunity cost approach given their complementary nature. This synergistic approach demonstrates the importance of examining both residents' micro- and macro-level perspectives. This research has important implications for future event hosts by providing rigorous evaluation methods to provide a foundation for good governance and decision making to maximise the social value of events for host communities. This is important given the decreasing interest in bidding for and hosting mega-events (7), coupled with host community concerns around corruption and lack of transparency leading to an increasing reluctance of communities with respect to event hosting (8).

We would like to thank the contributors to this special issue, who, in their own way helped our vision of a broad-ranging curation of articles which would bring to the fore a range of perspectives, issues and themes across sport, leisure, tourism and events to illustrate various ways we can disrupt thinking and doing to arrive at better solutions into the future. Together, these articles highlight the social and well-being potential of sport, leisure, tourism and events and testify to the resonance of interdisciplinarity and transdisciplinary approaches that lay the groundwork for further research in these fields.
